# Adoption of an Evidence-Based Intervention for Mammography Screening Adherence in Safety Net Clinics

**DOI:** 10.3389/fpubh.2021.748361

**Published:** 2021-11-04

**Authors:** Jennifer Holcomb, Gayla Ferguson, Isabel Roth, Gretchen Walton, Linda Highfield

**Affiliations:** ^1^University of Texas Health Science Center at Houston, Houston, TX, United States; ^2^University of North Carolina at Chapel Hill, Chapel Hill, NC, United States; ^3^School of Public Health, University of Texas Health Science Center at Houston, Houston, TX, United States

**Keywords:** adoption, evidence-based intervention, safety net, mammography, appointment adherence

## Abstract

Through an academic-community partnership, an evidence-based intervention to reduce mammography appointment no-show rates in underserved women was expanded to safety net clinics. The partnership implemented four strategies to improve the adoption and scale-up of evidence-based interventions with Federally Qualified Health Centers and charity care clinics: (1) an outreach email blast targeting the community partner member clinics to increase program awareness, (2) an adoption video encouraging enrollment in the program, (3) an outreach webinar educating the community partner member clinics about the program, encouraging enrollment and outlining adoption steps, and (4) an adoption survey adapted from Consolidated Framework for Implementation Research constructs from the Cancer Prevention and Control Research Network for cancer control interventions with Federally Qualified Health Centers. The development of academic-community partnerships can lead to successful adoption of evidence-based interventions particularly in safety net clinics.

## Introduction

Widespread adoption and implementation of evidence-based interventions (EBIs) is critical to improve health outcomes by increasing community demand and access to healthcare services. Unfortunately, widespread scale-up adoption of EBIs in public health practice remains elusive, especially with community and healthcare organizations that serve underserved communities (1–3). The purpose of this community case study is to document four strategies by an academic-community partnership to improve adoption and uptake of a mammography screening evidence-based intervention (EBI) in Federally Qualified Health Centers (FQHCs) and charity care clinics serving underserved women in the Greater Houston area.

## Background and Rationale

Breast cancer is the most common cancer in the United States and the second leading cause of cancer mortality among women in the United States, with roughly one in eight developing breast cancer during their lifetime (4). Substantial breast cancer-related morbidity and mortality disparities persist among underserved communities including those who are uninsured and African American women (4). These breast cancer-related disparities are attributed to a lack of access to mammography screening services, an increased likelihood of a later stage cancer diagnosis, and lack of culturally appropriate care across the cancer continuum of care (5–8). Knowledge and beliefs about both breast cancer and mammograms' impact a woman's willingness to be screened (5). Fear of having the screening test performed and the possibility of pain or discomfort can deter underserved women from receiving a mammogram (6, 7). There are also women that would rather not know about the presence of cancer in their bodies (6, 7). In addition to cognitive barriers, underserved women report missing scheduled appointments due to logistical barriers, lack of transportation, child care, and the ability to take time from work (5, 6). While the average no-show rates from mammogram appointments is 6.2%, African American women who make up a good portion of underserved women, have a no-show rate 2.6 times higher than the 6.2% on average (9).

FQHCs and other safety net clinics in the United States are uniquely situated to reach women in the most need for breast cancer screening. More broadly, safety net clinics provide healthcare services to those who are uninsured or a Medicaid recipient, living in immigrant and minority comminutes, and those who are unable to afford health care services elsewhere. FQHCs are federally designated community-based safety-net providers that provide a comprehensive set of healthcare services including primary care and preventive care regardless of a patient's ability to pay (8, 9). Similarly, charity care clinics provides free or reduced cost care to patients without health insurance. Despite their importance, these clinics need assistance in adopting and implementing EBIs as many do not have staff with expertise or experience in this area or capacity to implement them on their own (8–10). Recent systematic reviews have identified some strategies shown to improve the adoption and scale-up of EBIs at clinical and system levels (11, 12). These include conducting educational meetings with clinical providers, developing incentive or penalty structures, and addressing human resource and cost barriers (11, 12). However, there is still much to learn in terms of which adoption strategies are feasible and most effective to support EBI scale up for breast cancer and other cancers, particularly in settings such as Federally Qualified Health Centers (FQHCs) that care for underserved communities.

Research shows complex health issues like breast cancer require a collaborative approach (13). Effecting positive change in the prevention of breast cancer requires patient support beyond an appointment referral or reminder from a health care provider. Creating a space for community organizations, clinical providers, and academic research entities to problem-solve as equal partners builds trust and facilitates the placement of appropriate EBIs. Collaboration and partnerships positively affect mammography screening behaviors and breast cancer disparities among underserved communities (13). Collaborations of organizations with complementary expertise has been shown to result in the creation of common goals, to fill health care gaps, and to promote efficiency (14).

### Context

The PMP intervention was implemented in FQHCs and charity clinics in the Greater Houston area through an academic-community partnership with The University of Texas Health Science Center at Houston (UTHealth) School of Public Health and the Breast Health Collaborative of Texas (BHCTexas) alongside three local mobile mammography providers who provided mammography screening assistance (15, 16). BHCT is a 501(c) (3) nonprofit statewide member network of over 600 individual and organizational breast health advocates. BHCT has a unique mix of members – ranging from breast cancer survivors, advocates whose lives have been touched by breast cancer, health care professionals working in breast health [e.g., clinicians, nurses, community health workers (CHWS), healthcare managers and administrators] and organizations providing breast health services. BHCT includes a member network of local FQHC and charity clinic members in the Greater Houston area who provide free or reduced cost care to underserved populations. The local clinic organizational members are represented by individual clinic leadership and staff at the frontlines of providing health care to underserved communities. This Collaborative is a member-driven organization and, as a unique organization in the state of Texas, unites the breast health community to educate, advocate, navigate and maximize resources with the vision of ensuring quality breast health care. BHCTexas administration and CHW staff had experience and expertise in collaborative leadership with extensive knowledge of the system of care for breast cancer in Texas and of issues facing underserved women in safety net clinics. This background made BHCTexas an ideal partner for facilitating intervention adoption. The academic researchers brought in BHCTexas as an equal partner to assist in the facilitation of intervention adoption into the mammography screening appointment process in the local FQHCs and charity care clinics comprising the BCHTexas member network.

### Peace of Mind Program (PMP) Programmatic Elements

The Peace of Mind Program (PMP) is an EBI adapted from a National Cancer Institute Research program to reduce mammography appointment no-show rates in underserved women (3, 15, 16). In the intervention, patient navigators provide tailored reminder phone calls based on the Transtheoretical Model of Change to counsel women through cognitive and system barriers to scheduled mammography appointments (3, 15, 16). In the development of the EBI, interviews and focus groups were conducted with African American women who had missed a mammography appointment within the last six months (17). The intervention was then tested within a mobile mammography practice setting with underserved African American women in in the Greater Houston Area (3). For the adoption of PMP in FQHCs and charity clinics, patients reviewed and provided feedback on reminder phone call scripts (15, 16). The previously developed intervention protocols were then adapted to include adoption and implementation strategies to support expansion to the FQHCs and charity clinics (15, 16).

### Conceptual Framework

A conceptual framework using selected constructs from across the five domains in the Consolidated Framework for Implementation Research (CFIR) and five phases from The International Association for Public Participation (IAP2) spectrum was created to guide stakeholder engagement with the safety net clinics in the PMP (16). The conceptual framework was used across adoption, implementation, and sustainment of the PMP. In terms of adoption, the researchers and BHCTexas recruited potential sites to PMP in the Inform IAP2 phase by first educating clinic staff about the strength and quality and relative advantage of the intervention. These activities targeted CFIR constructs in the Intervention Characteristics domain. In the Consult IAP2 phase, we sought feedback about the implementation climate and readiness of the clinic to adopt and implement PMP. These activities targeted CFIR constructs in the Inner Setting domain. The complete conceptual framework description has been reported elsewhere (16).

### Adoption Strategies

An adoption planning group – consisting of the academic research team and BHCTexas administration and CHWs – developed four adoption strategies that aligned with the Inform and Consult IAP2 phases in the stakeholder engagement conceptual framework (16, 18). The strategies included: (1) an outreach email blast targeting BHCTexas member clinics to increase program awareness, (2) an adoption video encouraging clinic enrollment in the program, (3) an outreach webinar educating BHCTexas members about the program, encouraging enrollment and outlining adoption steps, and (4) an electronic adoption survey adapted from CFIR constructs from the Cancer Prevention and Control Research Network (CPCRN) for cancer control EBIs with FQHCs (15, 19–22). A CPCRN work group developed a comprehensive set of measures to identify and promote the uptake of evidence-based approaches in cancer prevention and control (21, 22). The work group has reported the operationalization of the measures across CFIR constructs from all CFIR domains and their psychometric properties elsewhere (21). The measures across CFIR constructs demonstrated good discriminant validity and internal consistency when tested within the context of colorectal cancer screening in FQHCs (22). The survey consisted of 75 statement items measuring potential barriers and facilitators to intervention adoption across 12 constructs in three domains of the CFIR (See Supplementary Material for the full survey). For each survey statement, individual respondents rated the statement on a Likert scale from 5 (completely agreed with the statement) to 1 (completely disagreed with the statement).

The strategies reached the PMP target population through multiple modes. More than 50 people participated in the outreach webinar to receive continuing education units (CEUs). The adoption outreach video was sent to over 600 individual and organizational BHCTexas members and reach was tracked by Google Analytics to ensure members were viewing the video. The adoption survey was sent to all registered BHCTexas clinic members across Texas, which encompassed representatives of 20 local FQHC and charity care clinics providing free or reduced cost care to underserved communities, including mammography screening services, in the Greater Houston Area. These local clinics serve a diverse community of African American, Hispanic, Vietnamese, and white women between the ages of 40 to 64 years old, who were at or below 200% of the Federal Poverty Level for a family of four and who lacked health insurance. A total of 372 individual survey completion attempts were made including 50 attempts from the clinic leadership and staff at the 20 local clinics in the Greater Houston area. Out of the 43 local individuals who completed the question about employment, most had worked for the clinic for two years or less (34; 79%). Out of the 44 local individuals who completed the question about clinic role, most were clinic administrators (17; 39%) and CHWs/patient navigators (12; 27%). Out of the 20 total local clinics who took the adoption survey, 15 clinics adopted (75%) and five did not adopt (25%) PMP following exposure to the strategies.

Across the individual responses for each clinic, a mean score (M) between 1 and 5 was created to measure clinic member level of agreement with each statement item. Higher ratings of survey statements relating to the complexity of the intervention, trialability, and culture/stress in the clinic environment were associated with lower likelihood of adoption. The clinic members who did not adopt PMP had a higher level of agreement for complexity statement items such as, “It will be hard to train providers and staff to implement the PMP” (M = 2.8) and, “Using the PMP will require our clinic to make substantial changes to our way of doing things” (M = 3.3) compared with those who did adopt (respectively, M = 2.3 and 2.69). For trialability, those who did not adopt PMP had a higher level of agreement with survey statements such as, “Once we try the PMP it will not be easy to go back to our old way of doing things, even if we do not like it” (M = 3.6) compared with those who did adopt (M = 3.34). The clinic members who did not adopt had a higher level of agreement with survey statements relating to the stress-related cultural aspects present in the clinic environment than those who adopted: staff stress and strain (M = 3.1 vs. 3.06), heavy workload (M = 3.0 vs. 2.5), individual job stress (M = 2.5 vs. 1.90), and staff frustration (M = 3.3 vs. 2.21). On the other hand, cultural aspects such as openness, problem solving ability, job satisfaction, collegial trust and team spirit, in addition to a positive implementation climate and survey items measuring readiness for change, increased the likelihood of adoption in clinics. In regards to readiness for change, the clinic members who adopted had a higher level of agreement about survey statements relating to clinic leadership ensuring that there was time (M = 4.14 vs. 3.5) and systems (M = 4.34 vs. 3.7) in place to implement the PMP compared with those who did not adopt.

## Discussion

Several lessons emerged from this academic-community partnership, and the execution of adoption strategies to promote the uptake of an evidence-based intervention (EBI) in Federally Qualified Health Centers (FQHCs) and other safety net clinics:

- **Relationships are critical**. The research team leveraged established relationships to recruit clinics to participate in the PMP intervention. Initial meetings to talk about PMP adoption was a “warm” encounter with a trusted community organization. This helps with garnering commitment and addressing any reservations about working with the researchers or adopting the intervention.
- **Clinic input is key to successful adoption and implementation**. Though the researchers planned to randomly assign implementation start dates, it proved beneficial for sites to determine their start dates. With an accurate description of program and implementation requirements, sites were in the best position to determine when a successful launch was most likely to occur as they were most familiar with their current and future capacity to meet those requirements and had the autonomy to make necessary adjustments.
- **Staff turnover is pervasive**. A transfer of staff among both the clinic management and frontline implementers, the community partner, and the research team was observed. Since commitment to the program is vital to adoption, the loss of personnel that agree with adoption, support the program, and commit to providing time and system resources for the program decreases the likelihood of continued adoption. Initial adoption discussions should include upper management, middle management, and frontline implementers (i.e., patient navigators). Having commitment at multiple levels eases the threat of loss of commitment and establishes a culture of acceptance when staff turnover occurs.
- **An adoption survey can help researchers use time and resources more efficiently**. Researchers can focus efforts on prepared participants or identify concerns before continuing adoption discussions. The survey can help identify barriers and facilitators to adopting or implementing an intervention. The survey can also be used to identify positive and negative indicators for adoption in other interventions or scaling up a pilot program. In this case study, the adoption survey provided data on moderators to adoption and potential determinants that could be targeted in the development of post-adoption implementation strategies.

While this project was focused on the adoption and implementation of an EBI to improve mammography appointment adherence among underserved women in the Greater Houston Area, the implications of this work are relevant to community-academic partnerships and EBI adoption and implementation efforts in diverse settings and other health issues. Our experience in the adoption and implementation of the PMP intervention emphasized the importance of partnership, multiple engagement strategies, and flexibility throughout the research period. The academic-community partnership collectively allowed for open discussion, various communication methods and styles from which to share the program components, and multiple contacts for questions and clarity. Academic researchers, aided by BHCTexas administration and certified community health workers (CHWs), afforded the clinic staff a comfortable understanding of the EBI theory and methods and the logistics of the PMP intervention which aided in overall intervention adoption. The adoption, implementation, and sustainment of EBIs is often a years-long process, and staff turnover, unexpected life events, and natural disasters should be anticipated from the outset. To ensure success, broad collaboration, multiple engagement strategies, and commitment at multiple levels are key. Our experience using an adoption survey demonstrated early efforts to assess the implementation context, provide critical information on readiness to adopt an EBI, and might be useful tool in large-scale implementation efforts. While complex, development of academic-community partnerships can lead to successful uptake of EBIs to improve population health and reduce disparities in underserved communities.

## Data Availability Statement

Due to use of identifying information, only de-identified data may be requested. Data requests may be sent to the authors and will be considered upon reasonable request.

## Ethics Statement

The studies involving human participants were reviewed and approved by the Committee for the Protection of Human Subjects at The University of Texas Health Science Center at Houston (UTHealth). The Ethics Committee waived the requirement of written informed consent for participation.

## Author Contributions

LH conceived the study, led the study, oversaw data collection, analysis, contributed to manuscript writing and editing, and approved the final version. GW assisted with study design, led data collection in the field, and contributed to manuscript writing and editing. JH led manuscript development including data analysis. GF assisted with data analysis, manuscript writing, and program management. IR assisted with field data collection, data analysis, and manuscript writing.

## Funding

The author(s) disclosed receipt of the following financial support for the research, authorship, and/or publication of this article: This work was supported by the Agency for Healthcare Research and Quality (AHRQ) [Grant Number 1R18HS023255-01]; and work by IR, on this manuscript was supported by a T32 Fellowship from the National Center for Complementary and Integrative Health [Grant Number 5T32AT00378-14].

## Conflict of Interest

The authors declare that the research was conducted in the absence of any commercial or financial relationships that could be construed as a potential conflict of interest.

## Publisher's Note

All claims expressed in this article are solely those of the authors and do not necessarily represent those of their affiliated organizations, or those of the publisher, the editors and the reviewers. Any product that may be evaluated in this article, or claim that may be made by its manufacturer, is not guaranteed or endorsed by the publisher.
