# Supplementary material for: Adoption of an Evidence-Based Intervention for Mammography Screening Adherence in Safety Net Clinics
Source: Front Public Health. 2021 Nov 4;9:748361. doi: 10.3389/fpubh.2021.748361 (PMC8599425; doi:10.3389/fpubh.2021.748361)
Supplement: Supplementary file 1 [file Table_1.DOCX]

**Peace of Mind Program (PMP) Adoption and Implementation Survey**

**Mammography Intervention Characteristics (Intervention Characteristics Domain)**

***Relative advantage***

1. Using BHC Peace of Mind Program (PMP) will be more effective than our prior practices for increasing mammography appointment attendance rates
2. Using BHC Peace of Mind Program will provide an important benefit to our patients

***Complexity***

1. It will be hard to train providers and staff to implement the PMP
2. It will be hard to train someone on the staff to do the PMP telephone counseling
3. It seems like it will be pretty easy to keep the PMP program going
4. It will be complicated to implement PMP
5. Using the PMP will require our clinic to make substantial changes to our way of doing things
6. Using the PMP will require more work than our current funding will support

***Trialability***

1. Once we try the PMP it will not be easy to go back to our old way of doing things even if we do not like it
2. To try the PMP we will have to make systems changes that will be permanent
3. If we try the PMP and don’t like it, it will be pretty easy to change it or go back to our old practices

***Compatibility***

1. Using the PMP to increase mammography appointment attendance rates is compatible with current activities / practices in the clinic
2. Using the PMP to increase mammography fits well with the way I like to work
3. Using the PMP to increase mammography fits well with our current goals for women’s health
4. The PMP fits well with the way our providers like to do things
5. Using BHC Peace of Mind Program fits with our high priority to improve our breast health services

**Organization – Internal Environment (Outer Setting Domain)**

***Policies and Incentives***

1. Our health center is required to report mammography rates to an outside organization / funder?
2. Mammography rate is an important quality metric for our health center
3. During the past fiscal year, our health center received [additional] income / reimbursement attached to mammography rates or associated quality metrics
4. During the past fiscal year, our health center was recognized or rewarded for doing a good job with mammography (for example, public recognition; professional association recognition)

**Organization – Internal Environment (Inner Setting Domain)**

***Readiness for Change / Change Processes***

1. The clinic leadership will make sure that we have the time to implement the PMP
2. The clinic leadership will make sure that we have the systems in place to implement the PMP
3. Clinic leadership creates an environment in which new programs like the PMP can be accomplished
4. Someone at our clinic usually steps up to provide leadership to implement new programs (becomes a program champion to actively support and promote new programs)
5. Some of our staff (i.e., managers, supervisors, other staff), will become program champions, actively supporting and promoting the PMP beyond what is required.
6. Clinic staff takes an active interest in programmatic-related problems and successes.
7. Managers will actively support implementation of the PMP
8. Our clinic needs help to implement the PMP
9. Our clinic needs help to implement other evidence-based programs

***Patient Needs and Resources* (Outer Setting Domain)**

1. This clinic does a good job of assessing patient needs and expectations
2. Clinic staff promptly resolves patient complaints.
3. Patients' complaints are studied to identify patterns and prevent the same problems from recurring.
4. This clinic uses data on patient expectations and/or satisfaction when developing new services
5. This clinic uses data from patients to improve care.
6. This clinic does a good job of assessing patient needs and expectations regarding women’s health
7. This clinic does a good job of assessing breast health needs and expectations
8. Our clinic needs to improve breast health services to better meet the needs of patients
9. Our clinic needs to increase access to mammography to better meet the needs of patients
10. Our clinic needs to help patients become compliant with mammography screening recommendations

***Culture (General)***

1. At our clinic, people openly talk about what is and isn’t working
2. Most people in this clinic are willing to change how they do things in response to feedback from others.
3. After trying something new, we take time to think about how it worked
4. Most of the people who work in our clinic seem to enjoy their work
5. It is hard to get things to change in our clinic
6. Difficult problems are solved through face-to-face discussions.
7. People in this clinic operate as a real team
8. I can rely on the other people in this clinic to do their jobs well.
9. We regularly take time to reflect on how we do things.
10. Our clinic needs help to better communicate openly about what is and isn’t working.
11. Our clinic staff needs help to better communicate openly about what is and isn’t working regarding mammography services.
12. Our clinic staff needs help to work together to improve quality of mammography services.
13. Our clinic staff needs help to work together to implement
14. Most people in this clinic are willing to change how they do things
15. Throughout the clinic there is frequent and good communication about how different changes are going.
16. We use data to guide our clinic (e.g., performance reviews, assessments).

***Culture stress***

1. I am under too many pressures to do my job effectively
2. Our clinic staff members often show signs of stress and strain
3. The heavy workload in our clinic reduces our ability to successfully implement a program like PMP
4. Staff frustration is common in our clinic

***Culture effort***

1. People in our clinic always want to perform to the best of their abilities
2. People in our clinic are enthusiastic about their work
3. People in our clinic are enthusiastic about the PMP program
4. People in our clinic get by with doing as little as possible
5. People in our clinic do no put more effort into their work than they have to

***Implementation climate***

1. Clinic staff are expected to help PMP meet its goal (i.e., increase mammography appointment attendance rates)
2. Clinic staff will get the support they need to implement PMP
3. Clinic staff will get recognition for implementing PMP to increase mammography appointment attendance rates
4. Using PMP to increase mammography appointment attendance rates is a top priority for our clinic

***Available Resources***

1. In general, when there is agreement that change needs to happen in the clinic we have the necessary budget or financial resources
2. In general, when there is agreement that change needs to happen in the clinic we have the necessary training
3. In general, when there is agreement that change needs to happen in the clinic we have the necessary staffing
4. There are the equipment and materials to support the PMP in our clinic
5. There is enough patient awareness and need to support the PMP in our clinic
6. There is provider buy-in to support the PMP in our clinic
7. There is an implementation team to support the PMP in our clinic
